# Dr. John Dambach

**Published:** 1909-10

**Authors:** 


### OBITUARY

Dr. John Dambach, a graduate of the University of Buffalo, Medical Department, 1868, died at his home, No. 417 Michigan street, Buffalo, September 15, 1909. He was the father of Mrs. Pliny B. McNaughton and Mrs. Albert J. Beirlein.

Dr. Dambach had lived in his Michigan street home for many years and had built up a large practice on the east side.

